# Maternal and Neonatal Outcomes of Adolescent Pregnancy: A Narrative Review

**DOI:** 10.7759/cureus.25921

**Published:** 2022-06-14

**Authors:** Marvi V Maheshwari, Nabeeha Khalid, Pragnesh D Patel, Rahmah Alghareeb, Afshan Hussain

**Affiliations:** 1 Research, Our Lady of Fatima University College of Medicine, Valenzuela, PHL; 2 Cardiology, Omar Hospital and Cardiac Center, Lahore, PAK; 3 Research, St. George’s University School of Medicine, St. George’s, GRD; 4 College of Medicine, University of Baghdad, Baghdad, IRQ; 5 Research, Dow Medical College and Dr. Ruth K. M. Pfau Civil Hospital Karachi, Karachi, PAK

**Keywords:** low-birth-weight infants, adolescent motherhood, prematurity, maternal outcomes, neonatal outcomes, pregnancy in adolescence, fetal-maternal mortality, adverse pregnancy outcomes, teenage pregnancy, adolescent pregnancy

## Abstract

Adolescent pregnancy is the pregnancy of girls aged 10-19 years, leading to many maternal and neonatal adverse effects. These pregnancies have been a global concern for many decades and yet are still prevailing. This article has reviewed the significant determinants of adolescent pregnancy and various maternal adverse effects, including preeclampsia, preterm premature rupture of the membrane (PPROM), maternal anemia, sexually transmitted diseases, postpartum depression, and maternal deaths, and adverse neonatal outcomes, including low birth weight (LBW), prematurity, stillbirths, early neonatal demise, and low Apgar score. Various pathophysiologic events that lead to such adverse consequences have been briefly discussed in the article and how such occurrences can be overcome. This article has also emphasized the need to implement various modalities such as sex education, availability of contraceptives, and bringing community-level awareness to lower the prevalence of adolescent pregnancy.

## Introduction and background

Adolescent pregnancy, by definition, is pregnancy in girls between the ages of 10 and 19, where the majority are unintended pregnancies [1]. Approximately 15% of women below 18 years gave birth globally in 2015- 2020, and 90% or more of such deliveries occur in countries with low and middle income [1,2]. One in every five adolescent girls has given birth globally, and the risk rises to about one in every three adolescent girls in developing nations [3]. Although adolescent pregnancies are a global concern, for both developed and developing countries, according to the World Health Organization (WHO), approximately 21 million girls of age 15-19 get pregnant annually. Of these, 12 million give birth. About 5.6 million ended up with abortion, of which 3.9 million are reported as unsafe in the developing regions of the world, hence inclining the global burden more toward developing countries of the world [4]. Sub-Saharan Africa leads the charts of adolescent pregnancies compared to European and North American nations [2]. Early marriage, substance abuse, sexual violence, lack of availability of contraceptives, relatives with a history of adolescent birth, early sexual activity, lack of health services, limited maternal education, poverty, lack of parental support, child of a broken family, religious beliefs, lack of financial autonomy, social media, and pornography are among few of the risk factors for adolescent pregnancy [4,5].

**Figure 1:**
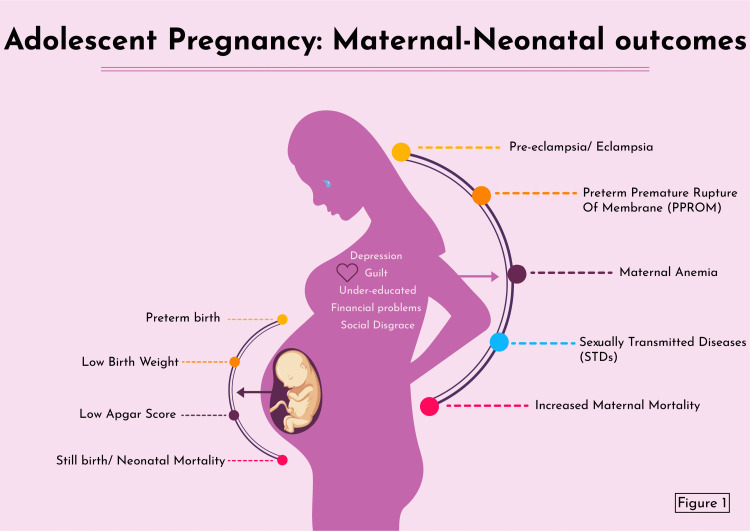
Adverse maternal and neonatal outcomes of adolescent pregnancy Image credits: Marvi Maheshwari

Girls with teen pregnancy are at increased risk of preeclampsia, preterm premature rupture of the membrane (PPROM), increased incidence of pregnancy-induced hypertension, anemia, sexually transmitted diseases, operative vaginal deliveries (forceps/vacuum), postpartum depression, and maternal deaths (Figure 1) [6-8]. Apart from the medical perspective, pregnant adolescent girls also suffer from guilt, financial constraints, inability to continue education, and disgrace from society [9]. Adverse neonatal outcomes, such as low birth weight (LBW), prematurity, stillbirths, early neonatal demise, small for gestational age, Apgar score at five minutes of <7, and various congenital anomalies, are expected among adolescent pregnant women (Figure 1) [1,7,10,11]. These pregnancies can be reduced by providing sex education, easy accessibility to contraceptives, the use of condoms, and reducing marriage before the age of 18 [3,11].

Pregnancies in adolescence result in no biological, mental, or social maturation process and adversely affect maternal and fetal outcomes due to biological immaturity, insufficient antenatal care, malnutrition, bad habits, stress, and depression and anxiety [11]. The primary objective of this article is to have a greater understanding for future researchers of not only adverse maternal outcomes (preeclampsia, preterm premature rupture of the fetal membrane, anemia, sexually transmitted diseases, and maternal mortality) but also adverse fetal outcomes (preterm births, low birth weight, Apgar scores, stillbirths, and neonatal mortality) concerning adolescent pregnancy.

## Review

Adolescent pregnancy: determinants

Early initiation of sexual activity and marriage at an early age with a partner of older age increases the likelihood of conception in the absence of contraception among adolescents in stable relationships, marriage, or union compared to those who are not. The risk of adolescent pregnancy is also high due to a lack of sex education and family planning and a lack of the ability to put that knowledge into effect. Lower education status, low socioeconomic class, and poverty also increase the rates of such pregnancies. Sexual abuse, peer pressure to have sex, lower self-esteem, depression, lower knowledge of contraceptives, and substance abuse also increase adolescent pregnancies. Family factors such as the long-time absence of both parents, single-parent household, child of an adolescent mother, and lack of proper communication with parents are also important determinants of such pregnancies (Table 1) [11,12]. As per a facility-based quantitative cross-sectional study conducted by Beyene et al. over four months at Assosa General Hospital with 783 randomly selected teenage females, 20.4% had teenage pregnancy, where the first sexual contact and the median age for marriage were 16-17 years, and 46.7% had forced sexual activities. The study concluded that educational attainment, age, marital status, work status, household earnings, and the use of various contraception methods were significant determinants of teenage pregnancy, and open-ended communications with parents, health checkups at school, and allowing young women to work acted as protective factors for preventing adolescent pregnancy [13]. Another study conducted by Akanbi et al. in Nigeria using the 2018 Nigeria Demographic and Health Survey (NDHS) with a sample population of 8,448 teenage females who had experienced pregnancies also showed that 19% of girls in Nigeria aged 15-18 years had undergone pregnancy with significant risk factors for adolescent pregnancy being living with significant other (84.3%), uneducated (43.7%), had health checkups in one year (42%), knew about family planning (84.3%), 18-19 years of age (33.2%), living in villages (27.2%), and poverty-stricken (32%) [14]. All the included studies related to the major determinants of adolescent pregnancy have been listed in Table 2.

**Table 1:** Major determinants for adolescent pregnancy

Determinants
Marriage/stable relationship
Older-aged partner
Lack of sex education and family planning
Lower education status
Depression and low self-esteem
Poverty
Substance abuse
Peer pressure
Child of an adolescent mother
Single-parent household
Absence of both parents

**Table 2:** Summary of the studies included for the determinants of adolescent pregnancy

Reference	Type of study	Population selected for the study	Time frame of the study	Region	Conclusion
Akanbi et al. [14] (2021)		8,448 teenage females of age 15-18 years	2018	Nigeria	The determinants of pregnancy involve living with a significant other, having no education, fewer health checkups in one year, knowledge of family planning, 18-19 years of age group, living in a village, and poverty.
Beyene et al. [13] (2015)	Facility-based quantitative cross-sectional study	783 randomly selected teenage females	January to April 2014 (four months)	Assosa General Hospital, Ethiopia	Educational attainment, age, marital status, work status, household earnings, and the use of various contraception methods were the major determinants of teenage pregnancy. It was also noted that open-ended communications with parents, health checkups at school, and allowing young women to work acted as protective factors for preventing teenage pregnancy.

Adolescent pregnancy: maternal outcomes

Preeclampsia in Adolescent Mothers

Adolescent mothers are prone to preeclampsia, a progressive hypertensive disorder of pregnancy that can present with multiorgan involvement, leading to adverse maternal and perinatal consequences, particularly for primigravid adolescent females [15,16]. A prospective case-control study was conducted by Medhi et al. for one year (2014) in Northeast India in a tertiary care hospital; the study included a population size of 165 adolescent primigravid (15-19 years) who had completed 28 weeks of gestation with singleton pregnancy delivering in that institution and 330 adult primigravid (20-25 years) who delivered next to the adolescent female. It was concluded that adolescent women (11.52%) were more likely to have preeclampsia as compared to adult women (6.06%) [17]. A systematic review and meta-analysis conducted by Macedo et al. over 50 years, with 291,241 adolescents in 30 countries, concluded that the overall prevalence of preeclampsia/eclampsia was 6.7%, which was highly dependent on the sociodemographic status of the adolescent female [18]. Preeclampsia can be correlated secondary to an immature uterus and the lack of a regular ovulatory menstrual cycle, which can cause defective decidualization, leading to faulty deep placentation causing the remodeling of spiral arteries, eventually leading to preeclampsia [15]. Fetal delivery is the only definitive treatment for preeclampsia [19]. If preeclampsia occurs before 37 weeks of gestation, fetal prematurity can be a significant complication [19]. Vigilant screening and monitoring of signs and symptoms to avoid the severe complications of preeclampsia are required in women diagnosed with preeclampsia [19].

Adolescent Pregnancy and Preterm Premature Rupture of Membranes

Preterm premature rupture of membranes, by definition, is the rupture of membranes before labor, before 37 weeks of gestation [20]. The etiology of PPROM can be multifactorial, including racial and socioeconomic status, smoking, sexual activity, nutritional deficiencies, vaginal bleeding, cervical parameters, and genital tract infections [20]. Adolescent females are more prone to PPROM as they have immature uterine and cervical blood circulation, making them more prone to underdiagnosed or diagnosed infections leading to PPROM by increasing inflammatory markers such as interleukins and prostaglandins, leading to chorioamniotic and decidual inflammation [21]. Marković et al. conducted a prospective study over four years (2011-2014), including 300 pregnant women of age 13-35, with 150 women of age 13-19 in one group and the rest of the 150 women aged 20-35 in another group. All of them had a healthy pregnancy at the beginning but later developed PRROM and premature rupture of membranes (PROM). The findings of this study showed that adolescent females had significantly high PPROM. It is also noted that premature rupture of membranes at term deliveries was also high among adolescent females [22]. The diagnosis of PRROM can be made by direct speculum examination, observing the amniotic fluid leakage, nitrazine test, crystallography, and ultrasound with peculiarly low amniotic fluid index [23]. The use of antibiotics by the mother, the use of corticosteroids according to gestational age, the use of magnesium sulfate for fetal neuroprotection, the use of tocolytic medicines, and the optimal time and method of delivery can all help manage PPROM well [23].

Adolescent Pregnancy and Maternal Anemia

Anemia is defined by the WHO as a decrease in hemoglobin or red blood cells associated with reduced oxygen-carrying capacity. A pregnant patient can be classified as severely anemic if her hemoglobin is below 7 g/dL, moderately anemic if between 7 and 9.99 g/dL, and mildly anemic if less than 11 g/dL [24]. Pregnant adolescents have a greater risk of anemia as higher iron intake is essential for a particular state of rapid growth where major biological modifications are in process. This can lead to iron deficiency, resulting in physical and cognitive damage to both adolescents and fetuses [25]. As per a cross-sectional study conducted by Pinho-Pompeu et al. in Brazil over nine years (2005-2013) including pregnant women of age 10-19 years, it is noted that the prevalence of anemia in these women was 41.27% (189), of which 65.60% were mildly anemic, 33.86% were moderately anemic, and 0.52% were severely anemic [26]. The study also showed that 87.24% of teen women received treatment, and among those who did not receive treatment had preterm births, small-for-gestational age neonates, and stillbirths as an expected consequence [26]. As per the WHO, prophylactic supplementation of 40 mg of elemental iron is advised from the beginning of pregnancy to the three months after delivery to all pregnant women [27].

Adolescent Pregnancy and Sexually Transmitted Diseases

Gonorrhea, chlamydia, trichomoniasis, syphilis, hepatitis B, human immunodeficiency virus (HIV), herpes simplex virus 1 and 2, and human papillomavirus infections are a few of the common sexually transmitted infections (STIs) seen during pregnancy [28]. In one way or another, these STIs can be harmful to the mother and the fetus via vertical transmission [28]. Adolescents are particularly more prone to STIs due to a lack of early sex education, substance abuse, social and gender inequality, and false beliefs [29]. In a study conducted by Asavapiriyanont et al. at Rajavithi Hospital, Bangkok, for eight months with 121 pregnant teenage females, it was found that 28.1% suffered from STIs (chlamydia, 19.8%; gonorrhea, 1.7%; hepatitis B, 3.3%; trichomoniasis, 1.7%; herpes simplex virus, 0.8%; condyloma acuminata, 0.8%). No cases of syphilis or HIV were detected. Non-STIs such as bacterial vaginosis and candidiasis were also seen in considerable numbers in these patients, concluding that STIs, particularly chlamydia, are common among adolescent females [30]. All pregnant women <25 years of age during their first prenatal visit must be tested for hepatitis B surface antigen (HBsAg), syphilis, gonorrhea, and chlamydia, as many sexually transmitted diseases can be asymptomatic as recommended by the Centers for Disease Control and Prevention (CDC) [31]. Timely diagnosis and management of adolescents with STIs are highly recommended to avoid severe consequences to the mother and the fetus from the progression of the disease [28].

Increased Maternal Mortality With Adolescent Pregnancy

Pregnancy and childbirth problems are the most significant cause of mortality among girls aged 15-19, with low- and middle-income countries responsible for 99% of global maternal fatalities among women aged 15-49 [32]. Conde-Agudelo et al. conducted a cross-sectional study in Latin America with 854,377 Latin American women’s recorded database younger than 25 years over 18 years (1985-2003), and it was found that adolescents aged 15 or under were at greater risk of maternal and early neonatal mortality and anemia as compared to women aged 20-24 [33]. It was also noted that postpartum hemorrhage, puerperal endometritis, operative vaginal delivery, episiotomy, low birth weight, preterm delivery, and small-for-gestational age newborns were all more common in teenagers of all ages. Cesarean delivery, third-trimester hemorrhage, and gestational diabetes were lower in adolescent moms [33]. The major causes of maternal mortality were maternal hemorrhage, hypertensive disorders, preeclampsia and eclampsia, maternal peripartum sepsis, obstructed labor, ruptured uterus, and abortion-related death [34].

A summary of all the included studies related to adverse maternal outcomes of adolescent pregnancy is presented in Table 3.

**Table 3:** Summary of all the included studies related to adverse maternal outcomes of adolescent pregnancy

Reference	Type of study	Population selected for the study	Time frame of the study	Region	Conclusion
Macedo et al. [18] (2020)	Systematic review and meta-analysis	291,241 adolescents	50 years (1969-2019)	30 countries	The overall prevalence of preeclampsia/eclampsia was 6.7%, which was highly dependent on the sociodemographic status of the adolescent female.
Marković et al. [22] (2020)	Prospective study	300 pregnant women of age 13-35 (150 women of age 13-19 and 150 women of age 20-35)	Four years (2011-2014)	University Clinical Center Tuzla, Clinic for Gynecology and Obstetrics	Adolescent females had significantly high preterm premature rupture of membranes. It was also noted that premature rupture of membranes at term deliveries was also high among adolescent females.
Pinho-Pompeu et al. [26] (2017)	Cross-sectional study	Pregnant women of age 10-19 years	Nine years (2005-2013)	University of Campinas (UniCamp), Brazil	The prevalence of anemia in women was very high in adolescent women, with a greater number of women with mild anemia as compared to moderate and severe anemia.
Medhi et al. [17] (2016)	Prospective case-control study	165 adolescent primigravid (15-19 years) who had completed 28 weeks of gestation with a singleton pregnancy who delivered in that institution and 330 adult primigravid (20-25 years) who delivered next to the adolescent female	One year (2014)	Northeast India	Adolescent women were more likely to have preeclampsia as compared to adult women.
Asavapiriyanont et al. [30] (2016)		121 pregnant teenage females	Eight months (October 2006 to May 2007)	Rajavithi Hospital, Bangkok	More than one-fourth of the pregnant females had STIs, including chlamydia, gonorrhea, hepatitis B, trichomoniasis, herpes simplex virus, and condyloma acuminata with the highest number of cases belonging to chlamydia.
Conde-Agudelo et al. [33] (2005)	Cross-sectional study	854,377 Latin American women younger than 25 years	18 years (1985-2003)	Latin America	Adolescents aged 15 or under were at greater risk of maternal and early neonatal mortality.

Adolescent pregnancy: neonatal outcomes

Adolescent Pregnancy and Preterm Birth

Preterm, as defined by the WHO, is the birth of babies before 37 weeks of gestation, subcategorized based on gestational age as extremely preterm (<28 weeks), very preterm (28-32 weeks), moderate-to-late preterm (32-37 weeks) [35]. Prematurity in adolescent women is associated with a low number of prenatal visits, late onset of prenatal care, and low educational level [36]. Vale de Almeida et al. evaluated the association between teen pregnancy and prematurity in two years (2011-2012) using the data collected from the national survey of Brazil consisting of 23,894 postpartum women and their newborn infants and found out that younger adolescents had the highest risk of spontaneous prematurity compared to older adolescents. It was also found that preterm is a severe problem for maternal and child health [37]. Prematurity leads to acute respiratory, immunologic, gastrointestinal, central nervous system, vision, and hearing problems and long-term motor, cognitive, behavioral, hearing, visual, health, social-emotional, and growth issues [38]. According to WHO guidelines, to reduce the morbidity and mortality secondary to preterm birth, interventions should be provided to the mother, such as steroid injections before the delivery of the baby, antibiotics for the mother when the water breaks before the initiation of labor, and magnesium sulfate to prevent neurologic impairment of the baby later in future, as well as interventions for the newborn baby such as thermal care, kangaroo mother care, feeding support, safe oxygen use, and other treatments modalities to help the neonate breath [35].

Adolescent Pregnancy and Low-Birth-Weight Neonates

The WHO has defined low birth weight (LBW) as a birth weight of less than 2,500 g (up to and including 2,499 g) in a newborn. Very low birth weight (VLBW) (<1,500 g) and extremely low birth weight (ELBW) (<1,000 g) are two types of low birth weight [39]. Marvin-Dowle et al. studied the association between maternal and neonatal outcomes in adolescent women in a population-based cohort study over four years (2007-2010) among primigravid women aged ≤19 years (n=640) and 20-34 years (n=3,951) as a reference group in Bradford, Northern England. It was noted that extremely low birth weight was significantly higher in the adolescent group (≤19 years) compared with the reference group. It was also stated that very preterm and extremely preterm delivery were also higher in the adolescent group [40]. The determinants of LBW in adolescent pregnancy are nonacceptance of pregnancy, fewer than six prenatal consultations, not having standardized nutritional care, and preterm delivery [41]. LBW is highly associated with prenatal and neonatal mortality and morbidity and delayed cognitive development. An increased chance of chronic disorders later in life are all the risks related to pregnancy [42]. To reduce LBW prevalence, focus on early pregnancy identification, regular, readily available, and affordable prenatal care, enhancing maternal nutrition, addressing pregnancy-related disorders such as preeclampsia, and providing proper maternal care, perinatal clinical services, and social support is to be provided [39,43].

Adolescent Pregnancy and Low Apgar Score

Doctor Virginia Apgar developed the technique of Apgar scoring, also used as the acronym APGAR, appearance, pulse, grimace, activity, and respiration, which are the components of the score. This score is a quick way to assess newborns shortly after birth at one- and five-minute intervals and respond to resuscitation. Apgar scoring consists of factors involving color, heart rate, reflexes, muscular tone, and respiration. Cyanosis, hypoperfusion, bradycardia, hypotonia, respiratory depression, or apnea are symptoms of hemodynamic compromise, which can be determined by Apgar scoring. Each item is given a score of 0 (zero), 1, or 2, with scores of 7-10 considered good scores [44]. A low Apgar score is a more prevalent outcome of adolescent pregnancy than an adult counterpart secondary to different sociodemographic, obstetric, and nutritional factors [45]. Ogawa et al. studied the association between adolescent pregnancy and adverse outcomes in a multicenter cross-sectional study over six years (2005-2011) in Japan among 30,831 women under 25 years of age with a singleton pregnancy and noted that low Apgar scores were significantly higher in adolescent mothers as compared to women aged 20-24 years. They also noted that the association of preterm birth, low birth weight, and a small proportion of lower Apgar could also be secondary to low maternal height [46]. When compared to a hospital-based retrospective cohort study by Yadav et al. in Nepal of 4,101 deliveries to compare the outcomes between teenage (15-19) and adult pregnancies (20-29) over one year period (2005-2006), no significant difference was noted in low Apgar score among the two groups [47]. A low Apgar score can be linked to neonatal complications, including respiratory distress, feeding problems, hypothermia, and seizures [48]. According to population studies, low Apgar scores at five minutes are associated with mortality and may indicate a greater chance of cerebral palsy, but not necessarily with individual neurologic disability [44].

Adolescent Pregnancy and Stillbirths

According to the CDC, a stillbirth occurs when a baby dies or is lost before or during delivery. Stillbirth can be characterized as early stillbirth if the fetal death occurs between 20 and 27 weeks of pregnancy, late stillbirth if the fetal death occurs between the 28th and 36th week of pregnancy, or term stillbirth when the pregnancy is 37 weeks or longer. The fetus is lost before or during delivery [49]. As per a study conducted in Missouri, the risk of intrapartum stillbirth is four times and 50% higher in older adolescent pregnant females than in adult pregnant women [50]. In a cross-sectional study conducted in Hebei, China, by Zhang et al. worked over four years (2013-2017) with 238,593 women subdivided into the adolescent group (10-19) and adult group (20-34), adolescent women had a higher risk of stillbirth and neonatal death as compared to the adult group. It was also noted that preterm deliveries were at a higher rate in the adolescent group [51]. The biological immaturity of the young adolescent who is still developing can trigger fetal-maternal rivalry for nutrients as the pregnancy proceeds, hence jeopardizing fetal growth, development, and survival as the pregnancy progresses. Social factors also play a role in stillbirths [50]. Stillbirths can be avoided by proper family planning to prevent unintended pregnancies, good health and nutrition before and during pregnancy, and quality and respectful antenatal and childbirth care, including adequate qualified healthcare staff and midwives [52].

Adolescent Pregnancy and Neonatal Mortality

Neonatal mortality is the death of the newborn baby occurring during the first 28 completed days of life. Early neonatal deaths occur during the first seven days of life, and late neonatal deaths occur after seven days but before 28 days of life [53]. The neonatal mortality risk is high among infants born to mothers aged 12-15 years, which can in part be explained by differences in socioeconomic factors in younger versus older mothers; the risk is mediated primarily through preterm delivery, low birth weight, neonates being small for gestational age, and some interaction of these variables [54]. In a study conducted by Neal et al. using the data of 64 demographic and health surveys of 45 different nations collected between 2005 and 2015 to analyze the relationship between adolescent motherhood and neonatal mortality, it was noted that the risk of neonatal mortality for maternal age under 16 years old was more significant in all regions. Socioeconomic, health services, and demography did not play much of a role in reducing mortality [55]. Hence, strategies should be made to lower adolescent births to reduce neonatal mortality [55].

A summary of all the included studies related to adverse neonatal outcomes of adolescent pregnancy is presented in Table 4.

**Table 4:** Summary of all the studies included for adverse neonatal outcomes in adolescent pregnancy

Reference	Type of study	Population selected for the study	Time frame of the study	Region	Conclusion
Zhang et al. [51] (2020)	Cross-sectional study	238,593 women subdivided into the adolescent group (10-19) and the adult group (20-34)	Four years (2013-2017)	Hebei, China	Adolescent women had a higher risk of stillbirth and neonatal death as compared to the adult group.
Vale de Almeida et al. [37] (2020)		23,894 postpartum women and their newborn infants	Two years (2011-2012)	Brazil	Younger adolescents had the highest risk of spontaneous prematurity compared to older adolescents.
Ogawa et al. [46] (2019)	Multicenter cross-sectional study	30,831 women under 25 years of age with a singleton pregnancy	Six years (2005-2011)	Japan	Low Apgar scores were significantly higher in adolescent mothers as compared to women aged 20-24 years.
Neal et al. [55] (2018)		Adolescent mothers	10 years (2005-2015)	45 countries	The risk of neonatal mortality for maternal age under 16 years old was greater in all regions. Socioeconomic, health services, and demography did not play much of a role in reducing mortality.
Marvin-Dowle et al. [40] (2018)	Population-based cohort study	Primigravid women aged ≤19 years (n=640) and 20-34 years (n=3,951) as the reference group	Four years (2007-2010)	Bradford, Northern England	Extremely low birth weight was significantly higher in the adolescent group (≤19 years) compared with the reference group. It was also noted that very preterm and extremely preterm deliveries were also higher in the adolescent group.
Yadav et al. [47] (2018)	Retrospective cohort study	4,101 deliveries of teenage (15-19) and adult (20-29) pregnancies	One year (2005-2006)	Nepal	No significant difference was noted in low Apgar scores between the two groups.

Long-term consequences of adolescent pregnancy

The emotions of the families to the adolescent pregnancies range from outrage and disappointment to abandonment, silence, acceptance, and forgiveness. Suicidal thoughts, guilt, loneliness, worry, and stress are among the psychological concerns faced by several adolescent mothers. They also face difficulties returning to school, low socioeconomic level, and social stigmatization [9,56]. Teen moms are more prone to postpartum depression, and infants of teen mothers are more prone to developmental delays and behavioral issues later in life [56].

Steps to reduce the prevalence of adolescent pregnancy

Closing pornographic stores that accept minors, utilizing the law to punish rapists, involving the political head in a campaign against early pregnancies, school departure before dark, locally accessible schools, and providing education regarding readily available contraceptives such as condoms, patches, vaginal rings, birth control pills, and injectable birth control methods are few of the steps that are to be taken to reduce the prevalence of adolescent pregnancy [57,58]. According to the CDC, teenagers should be encouraged to avoid having sex at the healthcare level. Long-acting reversible contraception (LARC), such as intrauterine devices and implants, should be considered safe and effective for teenagers as birth control. Teenagers should have proper guidance regarding LARCs, and the benefits and adverse effects should be discussed. LARCs should be widely available, and physicians should be trained to insert and remove LARCs. Teenagers should be enlightened about STIs and how they still can be affected, so proper use of condoms should be encouraged [59].

Limitations of the study

All the significant consequences of adolescent pregnancy are mentioned in this study. Still, there can be a lot more to adolescent pregnancy that may be undiscovered yet or discovered, but not that frequently occurring, which is not brought to light by this article.

## Conclusions

Adolescent pregnancy is a global problem affecting almost all countries in the world. In this article, all the significant consequences of adolescent pregnancy leading to adverse maternal outcomes, such as preeclampsia, preterm premature rupture of membranes, anemia, sexually transmitted diseases, and maternal mortality, and adverse neonatal outcomes, such as preterm births, low birth weight, low Apgar scores, stillbirths, and neonatal mortality, are discussed after reviewing previously published studies. Future researchers and clinicians can use this article to get an idea about the depth of the problem of adolescent pregnancy and what significant maternal and neonatal adverse effects can occur during pregnancy. Various steps can be taken to avoid adolescent pregnancy, such as educating adolescents about contraception, including long-acting reversible contraceptives, sexual abstinence, reducing childhood marriages, and bringing community-level awareness about the problem. Despite the comprehensive knowledge about the prevalence of adolescent pregnancy and its adverse effects, it is still prevailing globally. Effective strategies need to be made globally to reduce the prevalence and the negative impacts of such pregnancies. More studies should be conducted to know in-depth other adverse maternal and neonatal effects to reduce the mortality of both mother and neonate.

## References

[1] Ganchimeg T, Ota E, Morisaki N (2014). Pregnancy and childbirth outcomes among adolescent mothers: a World Health Organization multicountry study. BJOG.

[2] (2022). UNICEF: Early childbearing. May.

[3] World Health Organization (WHO) (2022). World Health Organization: Adolescent pregnancy fact sheet. World Health Organization.

[4] (2022). World Health Organization: Adolescent pregnancy. https://www.who.int/news-room/fact-sheets/detail/adolescent-pregnancy.

[5] Bain LE, Muftugil-Yalcin S, Amoakoh-Coleman M, Zweekhorst MB, Becquet R, de Cock Buning T (2020). Decision-making preferences and risk factors regarding early adolescent pregnancy in Ghana: stakeholders' and adolescents' perspectives from a vignette-based qualitative study. Reprod Health.

[6] Kawakita T, Wilson K, Grantz KL, Landy HJ, Huang CC, Gomez-Lobo V (2016). Adverse maternal and neonatal outcomes in adolescent pregnancy. J Pediatr Adolesc Gynecol.

[7] Rexhepi M, Besimi F, Rufati N, Alili A, Bajrami S, Ismaili H (2019). Hospital-based study of maternal, perinatal and neonatal outcomes in adolescent pregnancy compared to adult women pregnancy. Open Access Maced J Med Sci.

[8] Dinwiddie KJ, Schillerstrom TL, Schillerstrom JE (2018). Postpartum depression in adolescent mothers. J Psychosom Obstet Gynaecol.

[9] Govender D, Naidoo S, Taylor M (2020). "I have to provide for another life emotionally, physically and financially": understanding pregnancy, motherhood and the future aspirations of adolescent mothers in KwaZulu-Natal South, Africa. BMC Pregnancy Childbirth.

[10] Serunjogi R, Barlow-Mosha L, Mumpe-Mwanja D (2021). Comparative analysis of perinatal outcomes and birth defects amongst adolescent and older Ugandan mothers: evidence from a hospital-based surveillance database. Reprod Health.

[11] (2022). World Health Organization: Sixty-fifth World Health Assembly. https://apps.who.int/gb/ebwha/pdf_files/WHA65/A65_13-en.pdf.

[12] Chung HW, Kim EM, Lee JE (2018). Comprehensive understanding of risk and protective factors related to adolescent pregnancy in low- and middle-income countries: a systematic review. J Adolesc.

[13] Beyene A, Muhiye A, Getachew Y (2015). Assessment of the magnitude of teenage pregnancy and its associated factors among teenage females visiting Assosa General Hospital. Ethiop Med J.

[14] Akanbi MA, Ope BW, Adeloye DO, Amoo EO, Iruonagbe TC, Omojola O (2022). Influence of socio-economic factors on prevalence of teenage pregnancy in Nigeria. African Journal of Reproductive Health.

[15] Brosens I, Muter J, Ewington L, Puttemans P, Petraglia F, Brosens JJ, Benagiano G (2019). Adolescent preeclampsia: pathological drivers and clinical prevention. Reprod Sci.

[16] Bokslag A, van Weissenbruch M, Mol BW, de Groot CJ (2016). Preeclampsia; short and long-term consequences for mother and neonate. Early Hum Dev.

[17] Medhi R, Das B, Das A, Ahmed M, Bawri S, Rai S (2016). Adverse obstetrical and perinatal outcome in adolescent mothers associated with first birth: a hospital-based case-control study in a tertiary care hospital in North-East India. Adolesc Health Med Ther.

[18] Macedo TC, Montagna E, Trevisan CM (2020). Prevalence of preeclampsia and eclampsia in adolescent pregnancy: a systematic review and meta-analysis of 291,247 adolescents worldwide since 1969. Eur J Obstet Gynecol Reprod Biol.

[19] Witcher PM (2018). Preeclampsia: acute complications and management priorities. AACN Adv Crit Care.

[20] Lee T, Silver H (2001). Etiology and epidemiology of preterm premature rupture of the membranes. Clin Perinatol.

[21] Fraser AM, Brockert JE, Ward RH (1995). Association of young maternal age with adverse reproductive outcomes. N Engl J Med.

[22] Marković S, Bogdanović G, Cerovac A (2020). Premature and preterm premature rupture of membranes in adolescent compared to adult pregnancy. Med Glas (Zenica).

[23] Meller CH, Carducci ME, Ceriani Cernadas JM, Otaño L (2018). Preterm premature rupture of membranes. Arch Argent Pediatr.

[24] World Health Organization (2022). World Health Organization: Haemoglobin concentrations for the diagnosis of anaemia and assessment of severity. World Health Organization.

[25] Sekhar DL, Murray-Kolb LE, Kunselman AR, Weisman CS, Paul IM (2016). Differences in risk factors for anemia between adolescent and adult women. J Womens Health (Larchmt).

[26] Pinho-Pompeu M, Surita FG, Pastore DA, Paulino DS, Pinto E Silva JL (2017). Anemia in pregnant adolescents: impact of treatment on perinatal outcomes. J Matern Fetal Neonatal Med.

[27] World Health Organization (2022). World Health Organization: Guideline: Daily iron and folic acid supplementation in pregnant women. World Health Organization.

[28] Cunningham FG, Leveno KJ, Bloom SL (2013). Sexually transmitted diseases. Williams obstetrics, 24th edition.

[29] Alfaro AC (2019). Adolescence and risk of sexually transmitted infections. J AIDS Clin Res Sex Transm Dis.

[30] Asavapiriyanont S, Chaovarindr U, Kaoien S, Chotigeat U, Kovavisarach E (2016). Prevalence of sexually transmitted infection in teenage pregnancy in Rajavithi Hospital, Thailand. J Med Assoc Thai.

[31] Workowski KA, Bolan GA (2015). Sexually transmitted diseases treatment guidelines, 2015. MMWR Recomm Rep.

[32] Neal S, Matthews Z, Frost M, Fogstad H, Camacho AV, Laski L (2012). Childbearing in adolescents aged 12-15 years in low resource countries: a neglected issue. New estimates from demographic and household surveys in 42 countries. Acta Obstet Gynecol Scand.

[33] Conde-Agudelo A, Belizán JM, Lammers C (2005). Maternal-perinatal morbidity and mortality associated with adolescent pregnancy in Latin America: cross-sectional study. Am J Obstet Gynecol.

[34] Neal S, Mahendra S, Bose K (2016). The causes of maternal mortality in adolescents in low and middle income countries: a systematic review of the literature. BMC Pregnancy Childbirth.

[35] (2022). World Health Organization: Preterm birth. https://www.who.int/news-room/fact-sheets/detail/preterm-birth.

[36] Martins Mda G, dos Santos GH, Sousa Mda S, da Costa JE, Simões VM (2022). [Association of pregnancy in adolescence and prematurity]. Rev Bras Ginecol Obstet.

[37] Almeida AH, Gama SG, Costa MC, Carmo CN, Pacheco VE, Martinelli KG, Leal MD (2020). [Teenage pregnancy and prematurity in Brazil, 2011-2012]. Cad Saude Publica.

[38] Institute of Medicine (US) Committee on Understanding Premature Birth and Assuring Healthy Outcomes (2007). Preterm birth: causes, consequences, and prevention. https://pubmed.ncbi.nlm.nih.gov/20669423/.

[39] World Health Organization (2022). International statistical classification of diseases and related health problems, 10th revision, second edition. World Health Organization.

[40] Marvin-Dowle K, Kilner K, Burley VJ, Soltani H (2018). Impact of adolescent age on maternal and neonatal outcomes in the Born in Bradford cohort. BMJ Open.

[41] Belfort GP, Santos MM, Pessoa LD, Dias JR, Heidelmann SP, Saunders C (2018). Determinants of low birth weight in the children of adolescent mothers: a hierarchical analysis. Cien Saude Colet.

[42] Institute of Medicine (US) Committee on Improving Birth Outcomes (2003). Improving birth outcomes: meeting the challenge in the developing world.

[43] Slap GB, Schwartz JS (1989). Risk factors for low birth weight to adolescent mothers. J Adolesc Health Care.

[44] Simon LV, Hashmi MF, Bragg BN (2022). APGAR score. In: StatPearls [Internet].

[45] Kassa GM, Arowojolu AO, Odukogbe AA, Yalew AW (2019). Adverse neonatal outcomes of adolescent pregnancy in Northwest Ethiopia. PLoS One.

[46] Ogawa K, Matsushima S, Urayama KY (2019). Association between adolescent pregnancy and adverse birth outcomes, a multicenter cross sectional Japanese study. Sci Rep.

[47] Yadav S, Choudhary D, Narayan KC, Mandal RK, Sharma A, Chauhan SS, Agrawal P (2008). Adverse reproductive outcomes associated with teenage pregnancy. Mcgill J Med.

[48] Thavarajah H, Flatley C, Kumar S (2018). The relationship between the five minute Apgar score, mode of birth and neonatal outcomes. J Matern Fetal Neonatal Med.

[49] (2022). Centers for Disease Control and Prevention: What is stillbirth?. https://www.cdc.gov/ncbddd/stillbirth/facts.html.

[50] Wilson RE, Alio AP, Kirby RS, Salihu HM (2008). Young maternal age and risk of intrapartum stillbirth. Arch Gynecol Obstet.

[51] Zhang T, Wang H, Wang X, Yang Y, Zhang Y, Tang Z, Wang L (2020). The adverse maternal and perinatal outcomes of adolescent pregnancy: a cross sectional study in Hebei, China. BMC Pregnancy Childbirth.

[52] (2022). World Health Organization: Stillbirth. https://www.who.int/health-topics/stillbirth.

[53] (2022). World Health Organization: Neonatal mortality rate (0 to 27 days) per 1000 live births) (SDG 3.2.2). https://www.who.int/data/gho/indicator-metadata-registry/imr-details/67.

[54] Sharma V, Katz J, Mullany LC (2008). Young maternal age and the risk of neonatal mortality in rural Nepal. Arch Pediatr Adolesc Med.

[55] Neal S, Channon AA, Chintsanya J (2018). The impact of young maternal age at birth on neonatal mortality: evidence from 45 low and middle income countries. PLoS One.

[56] Goossens G, Kadji C, Delvenne V (2015). Teenage pregnancy: a psychopathological risk for mothers and babies?. Psychiatr Danub.

[57] Nabugoomu J, Seruwagi GK, Hanning R (2020). What can be done to reduce the prevalence of teen pregnancy in rural Eastern Uganda?: multi-stakeholder perceptions. Reprod Health.

[58] Centers for Disease Control and Prevention. (2020, August 13 (2022). Centers for Disease Control and Prevention: Contraception. https://www.cdc.gov/reproductivehealth/contraception/index.htm.

[59] (2022). Centers for Disease Control and Prevention: Preventing teen pregnancy. https://www.cdc.gov/vitalsigns/larc/index.html.

